# Prevalence and Genetic Characteristics of Human Bocaviruses Detected in Patients with Acute Respiratory Infections in Bulgaria

**DOI:** 10.1155/2021/7035081

**Published:** 2021-11-15

**Authors:** Neli Korsun, Svetla Angelova, Ivelina Trifonova, Silvia Voleva, Iliana Grigorova, Iren Tzotcheva, Sirma Mileva, Penka Perenovska

**Affiliations:** ^1^National Laboratory “Influenza and ARI”, National Center of Infectious and Parasitic Diseases, 44A Stoletov Blvd, Sofia, Bulgaria; ^2^Pediatric Clinic, University Hospital Alexandrovska, Medical University, 1 Georgi Sofiiski Str, Sofia, Bulgaria

## Abstract

Нuman bocaviruses (hBoVs) are often associated with acute respiratory infections (ARIs). Information on the distribution and molecular epidemiology of hBoVs in Bulgaria is currently limited. The objectives of this study were to investigate the prevalence and genetic characteristics of hBoVs detected in patients with ARIs in Bulgaria. From October 2016 to September 2019, nasopharyngeal/oropharyngeal swabs were prospectively collected from 1842 patients of all ages and tested for 12 common respiratory viruses using a real-time RT-PCR. Phylogenetic and amino acid analyses of the hBoV VP1/VP2 gene/protein were performed. HBoV was identified in 98 (5.3%) patients and was the 6^th^ most prevalent virus after respiratory-syncytial virus (20.4%), influenza A(H1N1)pdm09 (11.1%), A(H3N2) (10.5%), rhinoviruses (9.9%), and adenoviruses (6.8%). Coinfections with other respiratory viruses were detected in 51% of the hBoV-positive patients. Significant differences in the prevalence of hBoVs were found during the different study periods and in patients of different age groups. The detection rate of hBoV was the highest in patients aged 0–4 years (6.9%). In this age group, hBoV was the only identified virus in 9.7%, 5.8%, and 1.1% of the children diagnosed with laryngotracheitis, bronchiolitis, and pneumonia, respectively. Among patients aged ≥5 years, hBoV was detected as a single agent in 2.2% of cases of pneumonia. Phylogenetic analysis showed that all Bulgarian hBoV strains belonged to the hBoV1 genotype. A few amino acid substitutions were identified compared to the St1 prototype strain. This first study amongst an all-age population in Bulgaria showed a significant rate of hBoV detection in some serious respiratory illnesses in early childhood, year-to-year changes in the hBoV prevalence, and low genetic variability in the circulating strains.

## 1. Introduction

Acute respiratory infections (ARIs) are associated with a huge number of disease cases, outpatient visits, and hospitalizations and significant medical and social costs, thus representing a significant burden on healthcare systems and society as a whole. A wide range of viruses from different families cause respiratory tract diseases of varying severity. Human bocaviruses (hBoVs) are relatively recently identified respiratory pathogens belonging to the family Parvoviridae, subfamily Parvovirinae, and genus *Bocaparvovirus* [1, 2]. They are small (20 nm) viruses with a nonenveloped icosahedral capsid and a nonsegmented, single-stranded, negative-sense or positive-sense DNA genome with a length of approximately ∼5.3 kb organized into three open reading frames (ORFs), which encode the nonstructural proteins (NS1-4), the nuclear phosphoprotein NP1, and the structural proteins VP1/VP2, respectively. The two capsid proteins are responsible for the binding to cellular receptors and transporting the genome to the cell nucleus, and they are the main targets for the host immune response. These proteins share a common C-terminal region of 542 amino acids (aa) and differ only in the N-terminal region of VP1 (VP1u) that consists of 129 aa and is the most variable region of the genome [3]. Based on phylogenetic analysis, hBoVs have been divided into 4 genotypes, among which hBoV1 is associated most frequently with respiratory tract illnesses, while hBoV2-4 are mainly pathogens of the gastrointestinal tract [4]. HBoV1 occurs in people of all ages, but is most commonly detected in young children with respiratory symptoms. Seroepidemiological studies show that, over 90% of 4-year-olds have antibodies to hBoV1 [5, 6]. Reinfections occur throughout life, which explains the high seroprevalence in older individuals. The detection rate of hBoV-DNA in patients with ARI varies from 1.5% to 33% in different countries and studies [3, 7]. HBoVs have been characterized by an unusually high incidence (up to 90%) of coinfections [8, 9]. In hBoV-positive samples, other respiratory viruses or bacteria are often detected, which calls into question the true role of hBoV1 as a respiratory pathogen. Despite the fact that hBoV1 is detected in asymptomatic individuals and is a frequent participant in coinfections, this virus has been accepted in a large number of studies as a true cause of upper and lower respiratory tract infections, including infections among hospitalized patients [7, 10, 11].

So far, information on the distribution and clinical significance of hBoVs in Bulgaria is very limited. Previous studies examining the prevalence of respiratory viruses in the country have focused on children less than 5 years old, and data are absent for the prevalence of hBoVs among patients over the entire age range. There were no data on the genetic characteristics of these viruses [12, 13]. The objectives of this study were to investigate the prevalence and circulation pattern of hBoVs among patients of all ages presenting with ARI symptoms and to perform genetic/amino acid sequence analyses of the VP1/VP2 gene/protein of hBoV strains.

## 2. Materials and Methods

### 2.1. Patients and Specimen Collection

Patients in the age range of 0–91 years treated for ARI in primary-care facilities or hospitals located in all 28 regions of the country were enrolled in the National Influenza Surveillance Program. ARIs were defined according to the ECDC (https://ecdc.europa.eu/en/infectious-diseases-public-health/surveillance-and-disease-data/eu-case-definitions). Nasopharyngeal and oropharyngeal specimens, both placed into a common sterile viral transport media tube, were prospectively collected from the enrolled patients either during a visit to the doctor or within the first 24 h of admission. The specimens were taken 1–7 days after the onset of the illness. After collection, swabs were stored at 2°C–8°C for up to 72 h and transported in ice packs to the National Laboratory “Influenza and ARD” which is recognized as a World Health Organization National Influenza Center. Specimens were processed immediately for virus detection or stored at −80°C prior to analysis.

### 2.2. Molecular Detection of Respiratory Viruses

Viral DNA and RNA were extracted using an ExiPrep Dx Viral DNA/RNA kit and ExiPrep16DX equipment (BioNeer, Daejeon, Republic of Korea) in accordance with the manufacturer's instructions. Respiratory samples were tested for influenza viruses using a real-time RT-PCR method and the SuperScript III Platinum One-Step qRT-PCR System (Invitrogen, ThermoFisher Scientific, Waltham, MA, USA). Primers and probes were provided by the International Reagent Resource (IRR), USA. Amplification was performed using a CFX96 thermal cycler (Bio-Rad Laboratories, Inc., Hercules, CA, USA) according to the protocol recommended by CDC-Atlanta, USA [14]. Clinical samples were screened for noninfluenza viruses including respiratory-syncytial virus (RSV), human metapneumovirus (hMPV), parainfluenza viruses (PIV) 1/2/3, rhinoviruses (RV), adenoviruses (AdV), and bocaviruses (BoVs) using singleplex real-time PCR assays and an AgPath-ID One-Step RT-PCR kit (Applied Biosystems, ThermoFisher Scientific, Waltham, MA, USA). The primers, probes, and PCR conditions used in the study were identical to those described previously [15, 16]. Positive and negative controls were included in each run. The RNAase-P gene served as an internal positive control for human nucleic acid. Clinical samples were tested in separate real-time RT-PCR assays for the RNAase-P gene, which provided verification of RNA integrity and the absence of PCR inhibition. For influenza type A and type B viruses, positive controls were provided by IRR, USA; for other targets, AmpliRun DNA/RNA Amplification Controls (Vircell, Granada, Spain) were used. The sequences of primers and probes, as well as thermocycling conditions, are shown in the supplementary table.

### 2.3. VP1/VP2 Gene Sequencing

Conventional PCR was performed to amplify a fragment of the hBoV VP1/VP2 gene region. Nucleic acid amplification was carried out using an Eppendorf Mastercycler instrument (Eppendorf, Stevenage, UK) and a Qiagen One-Step RT-PCR kit (Qiagen, Hilden, Germany) with primers/protocol described previously (Supplementary Table (available here)) [17]. The amplified products with a length of 576 base pairs (bp) corresponding to the nucleotide positions 3233–3808 in the genome of strain PK-5510 (accession number FJ170278) were analyzed by electrophoresis on 2% agarose gels stained with ethidium bromide. The amplicons were extracted and purified with a PureLink Quick Gel Extraction kit (Invitrogen, Thermo Fisher Scientific, Waltham, MA, USA) according to the manufacturer's instructions. The purified amplicons were sequenced in both directions using the BigDye™ Terminator v1.1 Cycle Sequencing Kit (Applied Biosystems, Thermo Fisher Scientific, Waltham, MA, USA) on an ABI 3730XL (Thermo Fisher Scientific) DNA Analyser.

Partial VP1/VP2 gene nucleotide sequences of hBoV strains analyzed in this study were deposited in GenBank under the accession numbers MW759050-MW759067.

### 2.4. Phylogenetic Analysis

The VP1/VP2 gene nucleotide sequences of representative strains of all known hBoV genotypes as well as sequences of human and animal parvoviruses were downloaded from GenBank using the Basic Local Alignment Search Tool (BLAST) (https://blast.ncbi.nlm.nih.gov/Blast.cgi). The sequences obtained in the present study were aligned with the published sequences using the MUSCLE program embedded in Molecular Evolutionary Genetics Analysis software (MEGA, version 6.06; https://www.megasoftware.net/). The best fit nucleotide substitution model, Tamura-3 parameter (T-92 + G), was determined using MEGA 6.06. A phylogenetic tree based on the VP1/VP2 gene was constructed using the maximum likelihood method within MEGA 6.06 software. The reliability of the tree topology was evaluated by bootstrapping with 1000 replications. The study strains were genotyped based on clustering with sequences representing known genotypes.

### 2.5. Deduced Amino Acid Sequence Analysis

Deduced partial amino acid sequences of Bulgarian hBoV strains were translated with the standard genetic code using MEGA software. To identify amino acid substitutions, the VP1/VP2 protein sequences of the study strains were aligned with the prototype St1 strain.

Putative N-glycosylation sites were predicted using the NetNGlyc 1.0 web server (https://www.cbs.dtu.dk/services/NetNGlyc) to identify the sequence motif N-X-S/T (sequon), where *X* can be any amino acid except proline. Only the sites with scores higher than 0.5 were accepted as glycosylated.

### 2.6. Statistical Analysis

Statistical analyses were performed using GraphPad Prism *v*. 8.4.1 (GraphPad Software, San Diego, CA, USA). Chi-square and Fisher's exact tests were used for analyzing the following categorical variables: patients' age, sex, clinical features of illness, and incidence of each virus. A *p* value of <0.05 was considered to be statistically significant.

## 3. Results

### 3.1. Patient Characteristics

This study was conducted from October 2016 to September 2019 and included three influenza seasons, each starting with week 40 of one year and ending with week 20 of the following year. A total of 1842 patients exhibiting symptoms of ARI were enrolled in the study: 498 in the first season, 500 in the second season, 743 in the third season, and 101 between seasons. Twelve percent (225/1842) of these patients attended outpatient healthcare centres, and 87.8% (1617/1842) were hospitalized. The patients' ages ranged from 10 days to 91 years (median age 4.2 years). In the target population, 1332 (72.3%) were 0–4 years old, 170 (9.2%) were 5–17 years old, 75 (4.1%) were 18–64 years old, and 265 (14.4%) were ≥65 years old. A total of 1007 (54.7%) of the patients were males, and 835 (45.3%) were females.

### 3.2. Viral Detection

**Figure 1:**
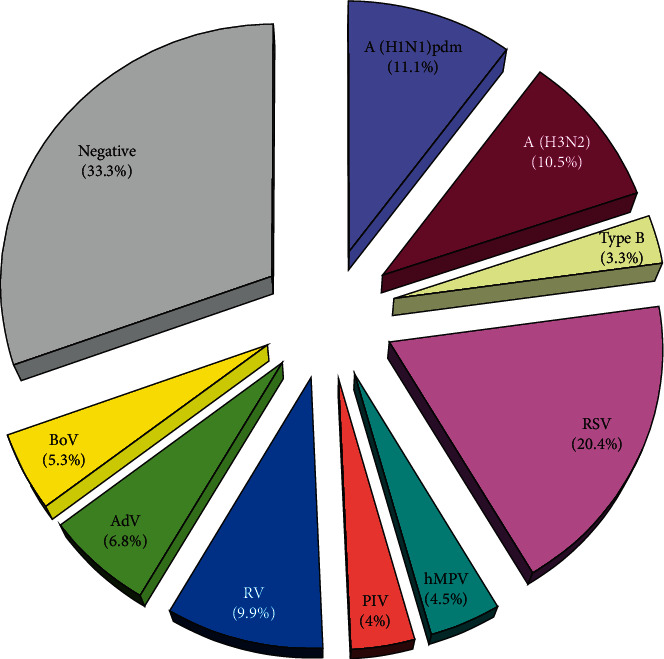
Distribution of different respiratory viruses detected in patients with ARI, Bulgaria, 2016–2019.

All the 1842 patients were tested for 12 respiratory viruses mentioned, and 1229 (66.7%) (130 outpatients and 1099 inpatients) of them were positive for at least one of these pathogens. Monoinfections were detected in 1042 (56.6%) patients; 172 (9.3%) patients were coinfected with two viruses; and 15 (0.8%) were coinfected with 3 viruses. HBoV was identified in 98 (5.3%) of the examined patients and was the 6^th^ most frequent virus among the respiratory viruses tested, following RSV (20.4%), influenza A(H1N1)pdm09 (11.1%), influenza A(H3N2) (10.5%), RV (9.9%), and AdV (6.7%) (Figure 1).

The detection frequency of hBoV in the first, second, and third winter seasons was 2.4% (12/498), 9.6% (48/500), and 3.8% (28/743), respectively, and in the periods between seasons, it was an average of 8.9% (9/101). The incidence rates of hBoV infection among the outpatients and inpatients were 4% (9/225) and 5.5% (89/1617), respectively, with no statistically significant difference (*p*=0.24) (Table 1).

**Figure 2:**
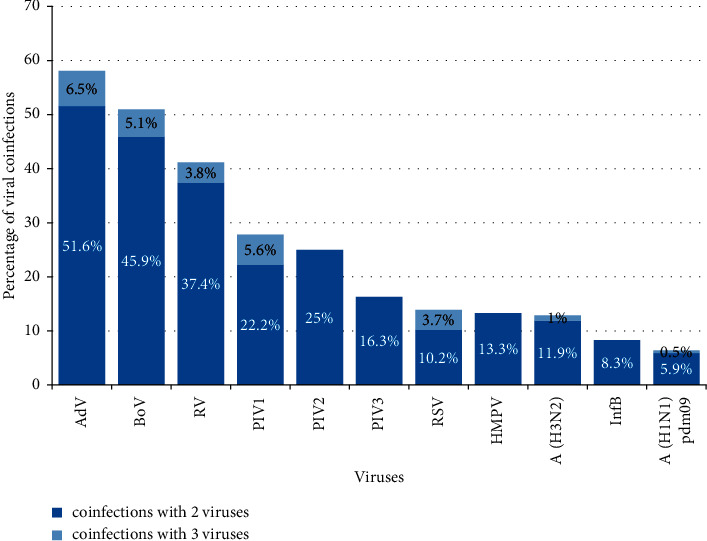
Distribution of identified respiratory viruses in cases of coinfections. The percentage of coinfections was calculated based on the total number of infections for each virus.

In total, 50 (51%) of the hBoV-positive patients were coinfected with one or two additional respiratory viruses. The most frequently identified copathogens were RVs (*n* = 17), followed by RSV (*n* = 11), AdV (*n* = 5), influenza A(H1N1)pdm09 (*n* = 4) and A(H3N2) (*n* = 3), PIV1 (*n* = 2), PIV3 (*n* = 2), and HMPV (*n* = 1). There were no cases of coinfections with PIV-2 and influenza type B. Codetection of two or three different viruses in the same clinical sample was found in 45 (45.9%) and 5 (5.1%) hBoV-positive patients, respectively. Among the respiratory viruses studied, AdVs, hBoVs, and RVs were characterized by the highest proportion of coinfections: 58.1%, 51%, and 41.2%, respectively (Figure 2).

**Figure 3:**
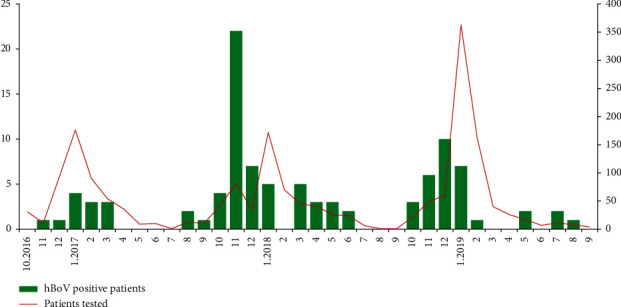
Monthly distribution of hBoV detections in Bulgaria, 2016–2019.

A higher frequency of hBoV infections was found between October and March (84.7% of all detections). HBoV was detected in few or no specimens in the summer months, but the number of samples tested during this period was relatively small. The highest number of hBoVs was identified in specimens obtained in November 2017 (22/81, 27.2%) and December 2018 (10/60, 16.7%) (Figure 3). The periods of increased activity of hBoV overlapped with those of rhinoviruses.

### 3.3. Distribution of Patients by Age and Sex

Viral respiratory infections were detected in 73.9% of patients aged 0–4 years, 57.2% of patients aged 5–17 years, 29.3% of patients aged 18–64 years, and 46.4% of patients aged ≥65 years. HBoV detection varied in patients aged between 3 months and 82 years. The incidence of hBoV infection was the highest among the youngest age group (0–4 years) (6.9%) and the lowest among the ≥65 years age group (0.4%). No hBoVs were detected in patients aged 18–64 years (Table 1). Children aged 0–4 years represented 72.3% of the patients studied, but accounted for 93.9% of the hBoV-positive cases (*p* < 0.05). In the age group of 5–17 years, three of the hBoV-positive patients were 5 years old and two were 6 years old. Among the patients ≥65 years of age, the only hBoV-positive patient was an 82-year-old man with serious breathing difficulties (emphysema). Coinfections of hBoV were detected only in children aged 0–4 years. Of the 98 hBoV-positive patients, 60 (61.2%) were males and 38 (38.8%) were females without a statistically significant difference (*p* = 0.21).

### 3.4. Clinical Characteristics

The contribution of hBoV and other tested respiratory viruses to the development of certain clinical syndromes, laryngotracheitis, bronchiolitis, pneumonia, and central nervous system (CNS) involvement (febrile seizures, cerebral oedema, aseptic meningitis, and encephalopathy), was analyzed. A total of 113, 346, 186, and 43 cases of laryngotracheitis, bronchiolitis, pneumonia, and CNS complications were diagnosed among the children aged 0–4 years, respectively. The hBoV was the second most commonly detected virus in patients with the diagnosis of laryngotracheitis (19/113, 16.8%), third in patients with the diagnosis of bronchiolitis (38/346, 11%), and sixth in patients with the diagnosis of pneumonia (9/146, 4.8%). The incidence of the hBoV as a single agent in children diagnosed with laryngotracheitis, bronchiolitis, and pneumonia was 9.7%, 5.8%, and 1.1%, respectively. Among the patients aged ≥5 years, hBoV alone was identified in 2.2% (2/91) of the cases of pneumonia (Table 2). No hBoV infections were detected in patients with neurologic complications.

### 3.5. Phylogenetic Analysis of hBoV

**Figure 4:**
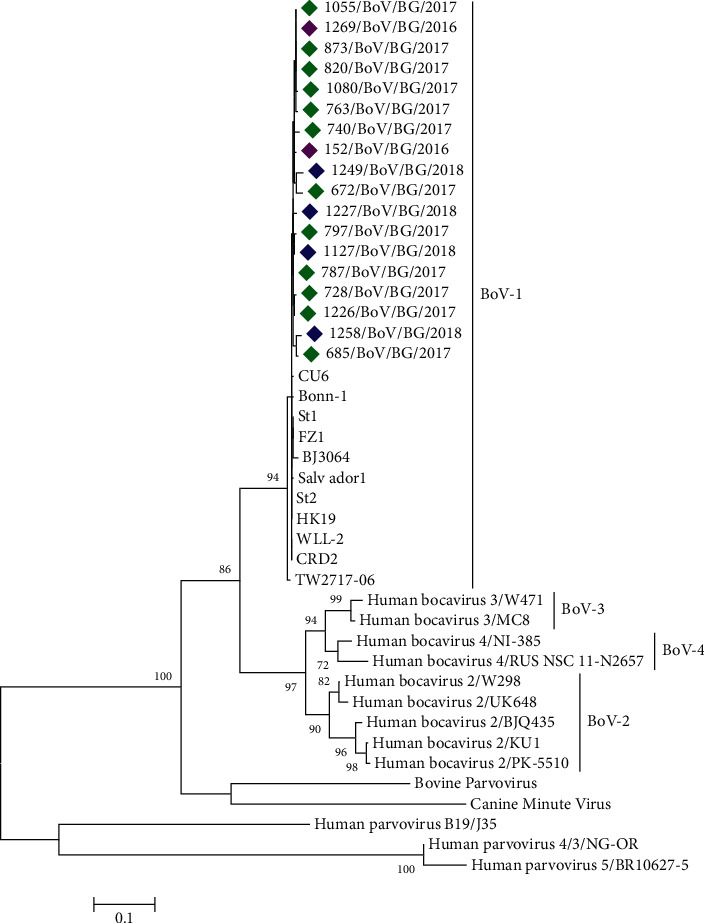
Phylogenetic tree based on a fragment of the VP1/VP2 gene of hBoV and other parvoviruses. Genetic distances were measured with the Tamura-3 model with gamma distribution (T92 + G). The phylogenetic tree was constructed with the maximum likelihood (ML) algorithm using MEGA software (version 6.0). The sequence of prototype hBoV strain St1 is shown in bold. The Bulgarian sequences, detected in 2016, 2017, and 2018, are shown by purple, green, and blue rhombuses, respectively.

For phylogenetic analysis, a fragment of the VP1/VP2 gene from 18 hBoV-positive clinical specimens was sequenced. The remaining positive samples had poor or failed PCR amplification or sequencing, probably due to the greater sensitivity of real-time PCR compared to conventional PCR for genotyping or due to low viral load in some samples. The hBoV sequences were obtained from hospitalized children aged 3 months–6 years with lower respiratory tract infections and from different regions of the country. Phylogenetic analysis showed that all hBoV strains isolated in this study belonged to the hBoV1 genotype and hBoV2–4 were not detected. The Bulgarian strains were grouped with the original strain St1 (GenBank access number DQ000495), identified by Allander et al. in 2005 (Figure 4). The hBoV1 sequences identified in this study showed 98.9%–100% homology at the nucleotide level and 97.8%–100% homology at the amino acid level.

#### 3.5.1. Deduced Amino Acid Sequence Analysis

**Figure 5:**
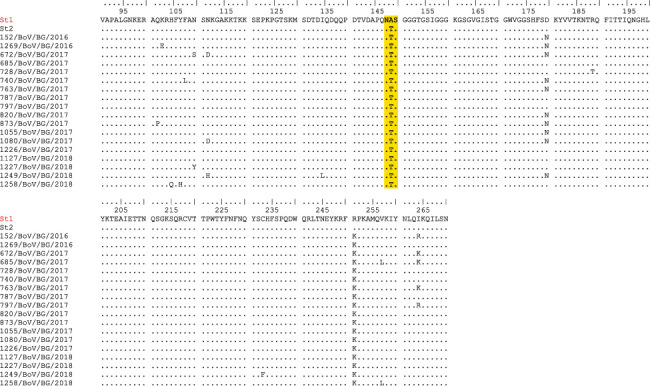
Partial amino acid sequences of the VP1/VP2 protein from Bulgarian hBoV strains. The alignment is shown relative to the sequence of the prototype St1 strain. Identical residues are identified as dots. Yellow shading highlights the predicted N-glycosylation site.

The deduced amino acid sequences of 18 Bulgarian hBoVs were aligned and compared with the sequence of the prototype strain St1 (Figure 5). In the VP1/VP2 protein, all Bulgarian hBoV sequences carried two amino acid substitutions: А149Т and R251 K. Ten (55.5%) sequences carried the substitution D180 N, and six (33.3%) sequences carried the substitutions I264 K/R. Single amino acid changes were found in 8 (44.4%) sequences in the area of the unique (VP1U) region, corresponding to the first 129 aa at the N-terminus of the VP1 protein. A total of 16.7% (3/18) of the Bulgarian sequences contained 2 amino acid substitutions relative to the prototype hBoV1 strain St1, 27.8% (5/18) sequences contained 3 substitutions, 38.9% (7/18) sequences contained 4 substitutions, and 16.7% (3/18) contained 5 substitutions in the studied fragment of the VP1/VP2 protein. All Bulgarian sequences harbored at least two substitutions compared to the St1 strain. The Bulgarian hBoV sequences analyzed possessed one potential N-linked glycosylation site, NTS, at positions 148–150.

## 4. Discussion

This study presents the prevalence and genetic characteristics of hBoVs amongst an all-age population in Bulgaria over three consecutive seasons, as well as the epidemiological and clinical features of hBoV respiratory tract infections. HBoV showed a moderate detection rate and was the 6th most prevalent virus among the tested 12 respiratory viruses. The general positive rate of hBoV was 5.3%, with a significant difference between seasons: 2.4%; 9.6%; and 3.8% (*p* < 0.05). Annual variations in the incidence of hBoV have also been observed in other countries [18]. The incidence rate of hBoV varies in different regions of the world depending on the sensitivity of diagnostic tests used, population studied, geographical location, climate, and time period of the study: 9.9% (Spain) [9], 12.1% (Italy) [19], 18% (the Netherlands) [20], 23.1% (Croatia) [21], 15.5% (Japan) [22], 6.33% and 11.64% (Argentina) [18], and 3.7% (South Africa) [23]. Previous studies in Bulgaria reported an hBoV prevalence of 7% among children younger than five years [12, 13, 24]. In this study, hBoVs were detected in similar proportions among the outpatients and inpatients (4% and 5.5%). A number of researchers have reported a relatively high incidence of hBoV infections among hospitalized patients [9, 25]. In the present study, significant differences were found in the prevalence of hBoV infection in patients of different ages. In close agreement with other reports, the positivity rate of hBoV was the highest in the youngest age groups (0–4 years), and 93.9% of all hBoV cases occurred in this age group [26]. The youngest child with identified hBoV was 3 months old, which suggests that infants younger than 3 months are probably protected from hBoV infection by maternal antibodies. No hBoVs were detected in patients aged 18–64 years. Similar to our data, in a large study performed in Kuwait between 2018 and 2020 with 5941 patients suffering ARI, none of the patients aged 10–29 years old had been infected with hBoV, and a small number of hBoV infections had been detected in the patients aged 30–64 [27]. The low incidence of hBoV infections in older children and adults, as shown in different studies, is probably due to life-long immunity acquired from primary hBoV infection during early childhood [1, 8]. In our study, no influence of the patients' gender on hBoV infection was observed in contrast to some reports, in which a higher prevalence of HBoV1 was detected in male patients [26]. Consistent with some reports, hBoV infection displayеd a clear seasonality with thе highest activity of the virus in the autumn and winter months [28, 29], while other authors report year-round circulation of hBoV [30, 31]. However, the smaller number of samples tested during the summer months, mainly from hospitalized patients, must be taken into account. The differences in the seasonality of hBoV infections are probably due to climatic factors. Information on the seasonal activity of respiratory viruses is important for strengthening surveillance and control measures to reduce the risk of nosocomial infections.

Previous studies have reported that hBoV has been associated with lower respiratory tract infections such as bronchiolitis, asthma exacerbation, and pneumonia [9, 29]. In this study, hBoV was the third and the sixth most common viral finding in children aged 0–4 years diagnosed with bronchiolitis and pneumonia, respectively. No association with CNS infections was established, although some authors report detections of hBoV1 in the cerebrospinal fluid of children with encephalitis [32].

An important feature of hBoVs, detected in Bulgaria, was their frequent involvement in coinfections. Codetection with other respiratory viruses was found in 51% of the hBoV-positive samples. A higher frequency of mixed infections could be expected if other respiratory viruses (e.g., coronaviruses) were also screened. A high rate of hBoV coinfections has been also found in studies in other countries: 75% (Spain) [9]; 78.1% (Argentina) [18]; 45.2% (Italy) [33]; and 66.7% (Vietnam) [34]. This fact can be explained by the prolonged shedding of hBoVs lasting for months after the primary infection [35], as well as by the frequent presence of these viruses in clinically healthy, asymptomatic individuals, which raises the question of whether hBoV are true pathogens of the respiratory tract or just accompanying viruses of other respiratory pathogens (opportunistic copathogens) [2]. In this study, hBoV was detected as a single viral agent in 9.7%, 5.8%, and 1.1% of the children aged 0–4 years suffering from laryngotracheitis, bronchiolitis, and pneumonia, respectively. Our results are in line with other studies, in which hBoV infection has been associated with severe respiratory diseases including bronchiolitis, pneumonia, and asthma exacerbation among young children [20, 31]. Zhou et al. have reported severe clinical manifestations and high viral loads of hBoV1 in the absence of other viral agents in previously healthy children [36]. There are reports that hBoVs are capable of causing even life-threatening illnesses [37, 38]. Furthermore, Brieu et al. have detected hBoV in hospitalized children (<5 years of age) with respiratory tract disease but in none of the children in the asymptomatic control group [39]. This finding indicates that hBoV is a true respiratory pathogen and not a harmless bystander.

Phylogenetic analysis showed that all the Bulgarian hBoV identified in the current study belonged to genotype 1 (hBoV1). The other three genotypes hBoV2–4 were not found, although some authors reported a low frequency of detection of these genotypes in respiratory samples [22, 40]. HBoV2–4 have mainly been identified in stool samples of patients with gastroenteritis and have been very rarely associated with respiratory infections. The current study involved only respiratory samples, which could explain the absence of the genotypes hBoV2–4. To investigate the divergence of the hBoV genome, a fragment of the variable VP1/VP2 gene of hBoV-positive samples was sequenced and the sequences of capsid proteins were compared with those of the prototype strain St1. The VP1/VP2 genes were highly preserved with minimal sequence variations among the isolates from the different years. No changing trend in the VP1/VP2 protein sequences was observed during the 3-year study period, although these surface proteins are potentially subjected to selective pressure of the immune response of the host. Weak glycosylation of capsid proteins and high degree of homology with the original hBoV strain St1 were found. The Bulgarian sequences contained 2–5 substitutions in the VP1/VP2 proteins compared to the St1 strain. These results are consistent with published data in other countries [19, 41–43]. Researchers from Italy reported that 7.6% of the 105 study strains had only one amino acid difference, 30.4% strains had two amino acid differences, and the remaining strains (61.9%) had at least three amino acid changes in comparison with the reference strain St1 [33]. The hBoV1 strains identified in Greece contain 5 amino acid substitutions in the VP1 protein, and one strain contains three additional substitutions [44]. Eight amino acid substitutions have been found in Cambodian VP1/VP2 sequences [31]. Based on the weak genetic and amino acid polymorphism, some authors suggest that hBoV infection occurs once in a lifetime because it causes the formation of life-long immunity based on the presence of neutralizing antibodies [45].

## 5. Conclusions

This first study amongst an all-age population in Bulgaria showed a significant frequency of hBoV detection in some serious respiratory illnesses in early childhood, year-to-year changes in the hBoV prevalence, and low genetic variability in the circulating strains. Our results could suggest a pathogenic role of this virus in cases of ARI, but additional methods (е.g., detection of hBoV-DNA and hBoV-specific IgM/IgG antibodies in serum samples) should be included to confirm the causality. Viremia, detection of HBoV1 IgM, and seroconversion of IgG antibodies have been considered by some researchers as diagnostic markers for acute hBoV1-induced respiratory illness [46]. Prolonged surveillance of circulating viruses is required to fully clarify the clinical significance of hBoV infection. Continuous genetic analysis of the detected hBoV would provide information on the genetic variations and molecular evolution of this infectious agent.

## Figures and Tables

**Table 1:** Distribution of hBoV-positive cases among outpatients and inpatients.

Age groups (years)	Outpatients (%) (*n*/*N*)	Inpatients (%) (*n*/*N*)	Total (%) (*n*/*N*)
0–4	4.4 (7/160)	7.3 (85/1172)	6.9 (92/1332)
5–17	8.3 (2/24)	2.1 (3/146)	2.9 (5/170)
18–64	0 (0/15)	0 (0/60)	0 (0/75)
≥65	0 (0/25)	0.4 (1/240)	0.4 (1/265)
Total	4.0 (9/225)	5.5 (89/1617)	5.3 (98/1842)

**Table 2:** Clinical diagnosis among the hBoV-infected patients according to age.

Age groups (years)	Total (%)	Clinical diagnosis				
		Laryngotracheitis (%)	Bronchiolitis (%)	Pneumonia (%)	Other respiratory syndromes (%)	CNS complications (%)
0–4	92 (93.9)	19 (20.7)	38 (41.3)	9 (14.1)	26 (28.3)	0
5–17	5 (5.1)	0	0	2 (40)	3 (60)	0
18–64	0	0	0	0	0	0
≥65	1 (1)	0	0	0	1 (63)	0
Total	98	19 (19.4)	38 (38.8)	11 (11.2)	30 (30.6)	0

## Data Availability

All data generated or analyzed during this study are included within the article (and its supplementary materials).

## Supplementary Materials

Supplementary Materials Supplementary Table. Primers/probes used in this study and thermocycling conditions. Abbreviations: NCR, noncoding region; F, forward primer; R, reverse primer; P, probe; FAM, 6-carboxyfluorescein; BHQ, black hole quencher; underlining and boldface indicate a locked nucleic acid.
