# Supplementary material for: Prevalence and Genetic Characteristics of Human Bocaviruses Detected in Patients with Acute Respiratory Infections in Bulgaria
Source: Int J Microbiol. 2021 Nov 15;2021:7035081. doi: 10.1155/2021/7035081 (PMC8608525; doi:10.1155/2021/7035081)
Supplement: Supplementary Materials — Supplementary Table. Primers/probes used in this study and thermocycling conditions. Abbreviations: NCR, noncoding region; F, forward primer; R, reverse primer; P, probe; FAM, 6-carboxyfluorescein; BHQ, black hole quencher; underlining and boldface indicate a locked nucleic acid. [file 7035081.f1.doc]

Supplementary Table. Primers/probes used in this study and thermocycling conditions

| Assays | Target gene | Primer/probe sequence (5'–3') | Final conc (nM) | Thermo  cycling conditions |
| --- | --- | --- | --- | --- |
| *Real time*  *RT-PCR for*  RSV detection | M | F: GGC AAA TAT GGA AAC ATA CGT GAA  R: TCT TTT TCT AGG ACA TTG TAY TGA ACA G  P: FAM-CTG TGT ATG TGG AGC CTT CGT GAA GCT-BHQ1 | 500  250  50 | RT at 45^0^C for 10 min, 94^0^C for 10 min, 45 cycles (94^0^C for 30 s and annealing at 60^0^C for 1 min) [15] |
| PIV-1 detection | HN | F: AGT TGT CAA TGT CTT AAT TCG TAT CAA T  R: TCG GCA CCT AAG TAA TTT TGA GTT  P: FAM-ATA GGC CAA AGA T(BHQ1)TG TTG TCG AGA CTA TTC CAA | 500  500  50 |
| PIV-2 detection | HN | F: GCA TTT CCA ATC TAC AGG ACT ATG A  R: ACC TCC TGG TAT AGC AGT GAC TGA AC  P: FAM-CCA TTT ACC T(BHQ1)AA GTG ATG GAA TCA ATC GCA AA | 750  750  50 |
| PIV-3 detection | HN | F: TGG YTC AAT CTC AAC AAC AAG ATT TAA G  R: TAC CCG AGA AAT ATT ATT TTG CC  P: FAM-CCC RTC TGT(BHQ1)TGG ACC AGG GAT ATA CTA CAA A | 750  500  200 |
| hMPV detection | F | F: CAA GTG TGA CAT TGC TGA YCT RAA  R: ACT GCC GCA CAA CAT TTA GRA A  P: FAM-TGG CYG TYA GCT TCA GTC AAT TCA ACA GA-BHQ1 | 600  600  100 |
| RV detection | 5’NCR | F: CP**A** GCC **T**GC GTG GC  R: GAA ACA CGG ACA CCC AAA GTA  P: FAM-TCC TCC GGC CCC TGA ATG YGG C- BHQ1 | 1000  1000  100 |
| AdV detection | Hexon | F: GCC CCA GTG GTC TTA CAT GCA CAT C  R: GCC ACG GTG GGG TTT CTA AAC TT  P: FAM-TGC ACC AGA CCC GGG CTC AGG TAC TCC GA-BHQ1 | 500  500  100 |
| BoV detection | NS1 | F: TGC AGA CAA CGC YTA GTT GTT T  R: CTG TCC CGC CCA AGA TAC A  P: FAM-CCA GGA TTG GGT GGA ACC TGC AAA--BHQ1 | 500  500  100 | 95°C for 3 min, 45 cycles (95°C for 15 s, 60°C 1 min) [16]  95^0^C for 35 s, 10 cycles (58^0^C for 1 min and 72^0^C for 1 min); 95^0^C for 30 s, 30 cycles (54^0^C for 45 s and 72^0^C for 45 s)[17] |
| *Conventional RT-PCR for* hBoV | VP1 | F: CGCCGTGGCTCCTGCTCT  R: TGTTCGCCATCACAAAAGATGTG | 500  500 |
|  |  |  |  |  |

**Abbreviations:** NCR, noncoding region; F, forward primer; R, reverse primer; P, probe; FAM, 6-carboxyfluorescein; BHQ, black hole quencher; Underlining and boldface indicate a locked nucleic acid.
